# Profile of children with cerebral palsy at a tertiary hospital in eastern Nepal

**DOI:** 10.1186/s12887-022-03477-x

**Published:** 2022-07-13

**Authors:** Shipra Chaudhary, Nisha Keshary Bhatta, Prakash Poudel, Jyoti Agrawal, Rosan Prasad Shah Kalawar, Jitendra Prasad Jayswal

**Affiliations:** 1 B P Koirala Institute of Health Sciences, Dharan, Nepal; 2 Janakpur Provincial Hospital, Janakpur, Nepal

**Keywords:** Cerebral palsy (CP), Clinical profile, Disability, Function, Spectrum

## Abstract

**Background:**

The clinical spectrum of Cerebral palsy (CP) can differ in various places depending upon knowledge of the people and resources for prevention, diagnosis and management. Although studied extensively in high-resource countries, adequate data related to CP from resource-constraint settings are lacking. This study aims to describe the profile of children with CP at a tertiary care center in eastern Nepal.

**Methods:**

This was a hospital-based cross-sectional descriptive study done from 2017 to 2018. Children 6 months to 15 years who presented with CP were enrolled and their clinical details recorded and described.

**Results:**

Amongst 110 children with CP, 74.54% were male. Majority (76.36%) were 5 years or below with the median age being 3(2.00–4.75) years. Children with spastic quadriplegia (44.44%) and Gross Motor Function Classification System level III (41.81%) were most common. Etiologically, perinatal factors (64.54%) like perinatal asphyxia (35.45%) and prematurity (20.90%) and postnatal infections (25.45%) were common. The common comorbidities were intellectual disability (71.81%) and epilepsy (66.36%). The main treatment modalities were: antiepileptics (59.09%) and centre-based physiotherapy sessions (35.45%). School education was provided in 23.07% with special education in 11.53%.

**Conclusions:**

This study describes the profile of CP at our centre in eastern Nepal. Predominance of perinatal complications and postnatal infections points towards the urgent need to further improve the perinatal and neonatal health care delivery system and practices.

**Supplementary Information:**

The online version contains supplementary material available at 10.1186/s12887-022-03477-x.

## Introduction

As per the latest definition by Rosenbaum et al., “Cerebral palsy (CP) describes a group of permanent disorders of the development of movement and posture, causing activity limitation, that are attributed to non-progressive disturbances that occurred in the developing fetal or infant brain. The motor disorders of cerebral palsy are often accompanied by disturbances of sensation, perception, cognition, communication, and behavior, by epilepsy, and by secondary musculoskeletal problems” [1]. It is the most common cause of motor disability in childhood [2].

The worldwide prevalence estimate of CP from population-based studies is 1 to 4 per 1000 live births [3]. Multiple risk factors are postulated in its etiology [4, 5]. Due to its heterogeneity and associated comorbidities, clinical classification of CP with a definite consensus is challenging [1, 6]. Currently, Gross Motor Function Classification System (GMFCS) is the most widely used functional classification worldwide [6, 7]. Clinical vigilance to early markers of CP and some costly investigations like neuroimaging may aid in early identification and prognostication [8, 9]. Early diagnosis and timely intervention with a comprehensive multidisciplinary approach play significant role in management of CP [10, 11].

There have been a lot of recent advances in the field of disability management worldwide but CP still remains an inadequately addressed problem in resource limited settings [12–16]. Its clinical spectrum may vary in different population depending upon the perception of people and resources for prevention, diagnosis and management [17–21].

Although shifted from low income to lower-middle income country since 2019, Nepal remains one of the poorest countries in Asia. According to the Human Development Report 2020, Nepal has Human Development Index (HDI) of 0.60 and Inequality adjusted HDI (IHDI) of 0.45 [22]. The national health care sector is progressing but at a very slow pace as compared to the developments worldwide. CP is still considered as a social stigma and there are limited studies related to CP from our country [18, 19]. Being a tertiary hospital in eastern Nepal, our centre has many children with CP presenting with various clinical features. Hence we felt a need to address these children and study their profile as a step forward in providing better care to these children.

## Methods

This was a hospital-based cross-sectional descriptive study done in the Department of Pediatrics & Adolescent Medicine, B P Koirala Institute of Health Sciences (BPKIHS), Nepal from 15th March 2017 to 14th March 2018. Written informed consent was collected prior to enrollment from all the parents or caregivers and assent from children, wherever applicable. Consent was obtained from all the parents or caregivers who were invited to this study, and there were no families who declined to participate. Those with neurodegenerative disorders, and disabilities due to other neurological causes besides CP were excluded.

CP was defined using the Rosenbaum et al. definition [1]. Detail history was taken from the mother or caregiver using a predesigned proforma followed by a complete physical, developmental and neurological examination done by pediatrician as per the standard practice. A copy of the proforma is available as supplementary material. Those having tone abnormalities and early markers or features of CP were also taken. CP was further classified by the pediatricians using Minear’s physiological and topographical classifications and GMFC as there was no consensus on the use of any specific classification system as per standard practice at our centre [6, 7, 23]. Risk factors were assessed clinically and with available documents and categorized as antenatal (before conception or birth), perinatal (during and immediate after birth) or postnatal (after birth). Common comorbidities like epilepsy, visual, hearing, speech, feeding, orthopedic problem, intellectual disability were assessed clinically by the pediatrician in history and examination and consultation with ophthalmologist, otorhinolaryngologist, orthopedician, psychologist along with investigations like Electroencephalogram, Visual Evoked Potential (VEP), Brainstem Evoked Response Audiometry (BERA) as required. Epilepsy was defined as at least 2 unprovoked (or reflex) seizures occurring more than 24 h apart as per International League Against Epilepsy (ILAE) 2014. Intellectual disability was defined according to Diagnostic and Statistical Manual of Mental Disorders (DSM- 5) with Intelligence Quotient (IQ) score of 70 or less. For visual problem, children were examined if they followed light (torch) and tested using visual acuity charts, cover-uncover test for strabismus and VEP. Similarly, hearing problem was assessed by BERA. Speech problem was considered if the children were not able to communicate verbally. Feeding problem was considered if the parent or caregiver had difficulty in feeding the children. Orthopedic problem was reported if there was contracture, kyphoscoliosis or hip dysplasia after expert consultation. Anthropometry was taken and interpreted using World Health Organization (WHO) and Centre for Disease Control (CDC) Growth Charts for children below and above 2 years respectively for assessing malnutrition as per WHO classification. Microcephaly was defined as a head circumference less than − 3 standard deviation (SD) below the average. Neuroimaging in form of Ultra sonogram cranium, Computed Tomography head or Magnetic Resonance Imaging (MRI) brain were done as per indication and feasibility and reports from the experts were noted.

All the enrolled children were managed and followed-up according to their specific needs, however, the study was not designed for long term follow up and no formally reported follow up occurred after a 3 months period. All data were entered in MS Excel and analyzed using SPSS 17.0 statistical software with simple descriptive statistics (mean, median, percentage).

## Results

In this study, there were 110 children with CP presenting to our hospital during the study period. Amongst them, majority (74.54%) were male. The age range of children enrolled in the study was 6 months to 15 years, with a median age of 3 years. Most children were aged 5 or below (76.36%). The mean age of identification of problem was 3.00 ± 1.23 years. Seventy six (69.09%) children had early markers and tone abnormalities suggestive of CP while 26 (23.63%) had more static and characterised CP and 8 (7.27%) were at risk of CP. The details of demographic and clinical characteristics are shown in Table 1.

**Table 1:** Demographic & clinical characteristics of children with cerebral palsy (*n* = 110)

Characteristic	No. (%) or Median (IQR)
**Gender**	
M/F	82/28 (74.54/25.46)
**Age**	
≤ 5 years	84 (76.36)
6–10 years	17 (15.45)
11–15 years	9 (8.18)
Median age	3(2.00–4.75)
**Physiological classification**	
Spastic	63 (57.27)
Dyskinetic	17 (15.45)
Ataxic	4 (3.63)
Mixed	26 (23.63)
**Topographical classification**	
Quadriplegia	28 (44.44)
Diplegia	22 (34.92)
Hemiplegia	12 (19.04)
Monoplegia	1 (1.58)
^**a**^**GMFCS classification**	
GMFCS I	3 (2.72)
GMFCS II	21 (19.09)
GMFCS III	46 (41.81)
GMFCS IV	31 (28.18)
GMFCS V	9 (8.18)

^a^*GMFCS* Gross Motor Function Classification System

The assessment of risk factors for CP showed that no definite risk factor for CP could be identified in 21 (19.09%) of children. The remaining 81% of children had at least one risk factor, with 24 children (21.81%) identified as having multiple risk factors. The majority of children had perinatal risks (64.54%), such as perinatal asphyxia (35.45%) and prematurity (20.90%), as shown in Table 2.

**Table 2:** Risk factors in children with cerebral palsy (*n* = 110)

Risk factors for CP	No. (%)
**Perinatal**	51 (64.54)
Perinatal asphyxia	39 (35.45)
Prematurity	23 (20.90)
Intra Uterine Growth Retardation (IUGR)	11 (10.00)
Jaundice	5 (4.54)
Neonatal hypoglycemia	1 (0.90)
Prolonged labour	4 (3.63)
**Postnatal**	29 (25.45)
Central Nervous System infection (meningitis)	20 (18.18)
Neonatal sepsis	16 (14.54)
**Antenatal**	11 (10.00)
Antepartum haemorrhage	7 (6.36)
Infertility treatment	2 (1.81)
Pregnancy induced Hypertension (PIH)	1 (0.90)
Alcohol exposure	1 (0.90)
**No identified risk factor**	21 (19.09)

**Fig. 1:**
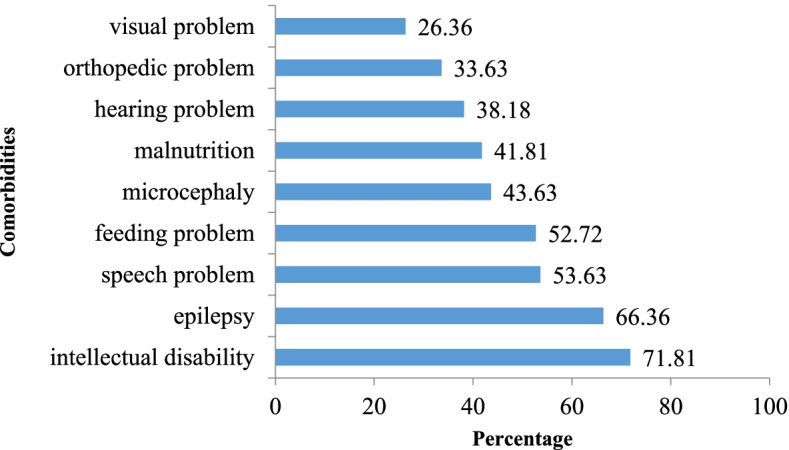
Comorbidities in children with cerebral palsy (*n* = 110)

Most of the children with CP had some form of co-morbidity. Figure 1 shows the various comorbidities found in our study.

Although many children couldn’t undergo investigations due to financial constraints, abnormalities were documented in 42 (68.8%) of the 61 children who underwent neuroimaging. The common neuroimaging abnormalities found were: cerebral atrophy (18), prolonged near total asphyxia involving deep grey matter (12), periventricular leukomalacia (4), basal ganglia calcification (3), thalamic infarct (2), white matter diffuse hyper intensities (2), and right parietal hemorrhage (1).

Our centre provided conservative symptomatic supportive therapy like medications for seizures and dystonia, physiotherapy, speech therapy, dietary and overall counselling. Sixty five (59.09%) children with CP were on anti-epileptics. Physiotherapy was advised to all but 71(64.54%) were practicing physiotherapy by themselves at home, 39 (35.45%) had received centre-based physiotherapy sessions, and 17 (15.45%) had both. There were 4 (3.63%) children using orthotic devices. Eleven (10%) children received speech therapy while none had received occupational therapy. Amongst 26 of 110 (23.63%) children of school age (> 5 years), 6 (23.07%) were receiving school education with special education in 3 (11.53%) children.

## Discussion

In our study of 110 children with CP presenting to a single tertiary hospital in eastern Nepal, majority of children were under 5 years with male predominance and underlying perinatal and neonatal risk factors. As this study had hospital-based convenience sample, majority had moderate to severe CP along with various comorbidities. Most children were managed with limited investigations and medications; comprehensive treatment focusing physiotherapy and special education were not adequate.

As our study lacked population based sample, the results might differ from the total population of children with CP in this region of Nepal. Another limitation of our study is the possibility of over diagnosis of CP due to inclusion of all the children from 6 months with early markers of CP and tone abnormalities suggestive of CP or at-risk of CP and not just confirmed CP in above 5 years. The diagnosis of CP in children under 5 years, although accepted and encouraged in recent studies, might not be accurate in 100% [24, 25]. Our study did not assess all the comorbidities by formal tests or screening questionnaires in all the children, thus missing some of the other possible comorbidities or underreporting of some of the evaluated comorbidities.

In our study, majority (74.54%) were male. Earlier studies from Nepal have also reported male predominance [18]. This correlates with the fact that males are more vulnerable to develop CP biologically and male/female ratio hasn’t changed significantly over time [26, 27]. Yet remarkably high sex ratio of 2.93 in our study as compared to the total population sex ratio of 0.98 and 1.02 in our country and globally respectively could be due to the practice of bringing male children for hospital care in the male-dominant society of our country [28].

Most children (76.36%) in our study were 5 years or below which is similar to other studies where most were under 5 years with maximum below 2 years in India and 2-5 years in Nepal [18, 19, 21]. These ages are also comparable to other countries like Nigeria and Uganda [29, 30]. It can be explained by the fact that CP manifests as motor delay affecting the attainment of milestones to a great extent and thus being noticed in this age group. The mean age of identification in our study was 3 ± 1.23 years. This is lower than average 5.5–6 years reported in earlier studies between 2008 to 2014 and could be an indication of increased awareness in our community and also due to the fact that we included all those at risk or with clinical features of CP [18, 19, 24, 25].

Spastic quadriplegia was the commonest type as reported in previous study from Nepal and Uganda [18, 30]. We had far less cases of hemiplegia (19.04% vs 40%) and diplegia (34.92% vs 46.4%) and far more quadriplegia (44.44% vs 13.6%) as compared to Sweden and Victoria [31]. The persistence of quadriplegia being common in our part could be explained by the underlying higher no. of perinatal asphyxia (35% vs 10%), lesser prematurity (21% vs 45%) and far more postnatal CP (33% vs 4%) in our part [4]. Likewise, perinatal, early neonatal and postnatal factors have been prevalent in resource-poor settings like Nigeria, Iraq, Malta and India [9, 21, 29]. Although recent studies show prenatal events to be more common these days, perinatal and postnatal complications still remain dominant in our population [4].This might be due to continuity of early marriage and childbirth, harmful cultural practices, lack of adequate or timely health services in our community.

Functionally, most (41.81%) of our children were GMFCS level III. This is comparable to the moderate severity reported in other studies [29, 30]. In a study of spastic CP in Iraq, Kareem AA reported significantly higher proportion of spastic diplegia and quadriplegia children having GMFCS level II & III and level IV & V respectively [32]. A larger proportion of children with GMFCS scores of IV or V were seen in Asian or African care centers compared with Australian, European or North American cohorts [33, 34]. Although the predominance of quadriplegic presentation supports the high no. of moderate to severe forms in our study, the far less mild cases (GMFCS I –II 22% vs 55%) could be due to missing of such cases as they might not have presented to hospital. A community or population based study needs to be done to get a clearer view of the functional distribution in our part.

The comorbidities found in our study were intellectual disability, epilepsy, speech, hearing, feeding, visual, orthopedic problems, malnutrition and microcephaly. Due to higher proportion of spastic quadriplegia, there were far more epilepsy and intellectual disability (2/3 vs 1/3) as compared to studies from high resource settings. Various studies have reported other comorbidities like learning disability, Attention Deficit Hyperactive Disorder (ADHD), Autism Spectrum Disorder (ASD), bowel, bladder problems, behavioral, emotional, sexual problems, pain and sleep problems in CP [14, 29, 30, 35]. The differences observed in the distribution of different co-morbidities may be due to various factors: lack of use of specific assessment tools, differences in risk factors, CP types, facilities and health-seeking behavior.

Though neuroimaging couldn’t be done in many of the children in our setting, the common abnormalities detected amongst the few were cerebral atrophy and prolonged near total asphyxia involving deep grey matter in contrast to periventricular leukomalacia or white matter injuries common in western world [9, 36]. This difference is justified by the predominance of perinatal asphyxia and spastic quadriplegia in our part. Yet, it is difficult to generalize the common findings in our set up due to the lack of availability/affordability of neuroimaging facilities in majority of the children.

Under the conservative management with symptomatic supportive therapy, there were far less children receiving center-based physiotherapy sessions (35.45% vs 98.00%) and special education (11.53% vs 17.20%) as compared to Sri Lanka [35]. The difference in physiotherapy may be due to lack of adequate manpower for physiotherapy in our center while the difference in education could be due to the differences in public knowledge/beliefs and socioeconomic factors. Since disability itself along with its comorbidities can have negative impact on school enrolment and continuity, therapy and education remain the mainstay in life-span approach to CP and hence need to be emphasized by all.

Although our study reflects the spectrum of CP at a tertiary hospital in eastern Nepal, the generalizability of the study results requires further large scale community-based studies with long term follow-ups. The predominance of intrapartum and post-natal antecedents in our setting definitely points towards the urgent need for some policy-level changes in global health management and more effective strategies in delivery of maternal and child health services in low resource settings.

## Conclusions

This study describes the profile of CP in our part. Predominance of perinatal complications and postnatal infections points towards the urgent need to further improve our perinatal and neonatal health care delivery system and practices. More of large-scale community based country or region-wise exploratory studies with long-term follow-ups are recommended at this stage for planning better strategies to address these problems in such settings.

## Supplementary Information

**Additional file 1.** Proforma.

## Data Availability

The datasets used and/or analyzed during the current study are available from the corresponding author on reasonable request.

## Abbreviations

BPKIHS: B P Koirala Institute of Health Sciences
CP: Cerebral Palsy
GMFCS: Gross Motor Function Classification System
MRI: Magnetic Resonance Imaging
