# Supplementary material for: Profile of children with cerebral palsy at a tertiary hospital in eastern Nepal
Source: BMC Pediatr. 2022 Jul 13;22:415. doi: 10.1186/s12887-022-03477-x (PMC9277817; doi:10.1186/s12887-022-03477-x)
Supplement: Supplementary file 1 — Additional file 1. Proforma. [file 12887_2022_3477_MOESM1_ESM.docx]

**Proforma**

**Name**

**DOB: …………………………………………… Age………………………. Gender: M/F Address……………………………… Contact no.:…………………………**

**Religion:**

**Ethnicity:**

**Mother’s name:**

**Mother’s age/education/occupation:**

**Father’s name:**

**Father’s age/education/occupation:**

**Family history significant: Y/N……………………………………………………………….**

**Pedigree chart:**

**Risk factors: Y/N**

Antenatal

APH/Intrauterine infection/PIH/ GDM/ Maternal disease: HTN /DM / Hypothryroidism /Hyperthyroidism // Teratogen exposure /Undernutrition/Iodine deficiency/ Smoking/Alcohol //Chronic disease/ Infertility treatment/ Fetal brain malformation/ other………………………………………………………………………………………..

Perinatal

Prematurity: Y/N (POG……………………………………………)

LBW: Y/N (B wt…………………………………………………………)

Prolonged labour: Y/N (Duration of labour:…………………………………………..)

Birth asphyxia: Y/N (Place: Hospital/Health care/Home; Type of delivery: SVD/AVD/CS; Cry: immediate/ delayed; APGAR:…………………………………………………………….)

IUGR: Y/N

Pathological Hyperbilirubinemia (requiring significant treatment): Y/N

Dyselectrolytemia/ Hypoglycemia/ Other: Y/ N

Postnatal

Neonatal sepsis: Y/N

CNS infection: Y/N

Head injury/ Hypoxic damage/ Genetic cause/ Other…………………………………………………

Child’s medical illness history…………………………………………………………………………………………..

**Developmental history (Gross motor/ Fine motor/ Social/ Language)……………………………………**

**Assessment:**

**Physical examination:**

Specific abnormality in vitals/ systemic examination:………………………………………………………

Anthropometry:

| **Observed** | **Expected** | **Interpretation** |
| --- | --- | --- |
| Weight for age |  |  |
| Length/Height for age |  |  |
| Weight for length/height |  |  |
| Mid upper arm circumference |  |  |
| Head circumference |  |  |

**Neurological examination:**

Cranial nerves abnormality if any…………………………………..

Motor

Bulk

Tone

Power

Reflexes

**Developmental age: (Gross motor…………./Fine motor…………/Social……………../Language………)**

**Early markers of CP : Y/N/NA (……………………………………………………………………………………………….)**

**Classification:**

| **Physiological** | **Spastic/ Dyskinetic/ Ataxic/ Mixed** |
| --- | --- |
| **Topographical** | **Quadriplegia/ Diplegia/ Hemiplegia/ Monoplegia** |
| **Functional (GMFCS)** | **Level I/ II/ III/ IV/ V** |

**Comorbidities:**

**Intellectual disability: Y/N (IQ=……………………….)**

**Seizure: Y/N**

**If Y: Type/ Frequency /Last episode/ AED/Control………………………………………………..........**

**Hearing problem (including abnormal BERA): Y/N**

**Visual problem (strabismus, nystagmus, abnormal VEP): Y/N**

**Orthopedic problem (deformity/contracture/gait): Y/N**

**Malnutrition: Y/N ……………………………..**

**Microcephaly: Y/N**

**Speech problem: Y/N**

**Feeding problem: Y/N**

**Behavior problem: Y/N**

**Others…………………………………………………………………..**

**Investigations:**

CT

MRI/MRS

USG cranium

EEG

TFT

BERA

VEP

Others

**School education: Y/N (If Y, Special education: Y/N)**

**Goals: (entry level- exit level)**

**Short term**

**Long term**

**Treatment:**

**Physiotherapy: Centre based/ Home based**

**AED: ………………………………………………………………………………………………………………………………….**

**Other medications:………………………………………………………………………………………………………………..**

**Comments**

**Follow-up**
